# Supplementary material for: Strawberry, Blueberry, and Strawberry-Blueberry Blend Beverages Prevent Hepatic Steatosis in Obese Rats by Modulating Key Genes Involved in Lipid Metabolism
Source: Int J Environ Res Public Health. 2023 Mar 1;20(5):4418. doi: 10.3390/ijerph20054418 (PMC10002361; doi:10.3390/ijerph20054418)
Supplement: Supplementary file 1 [file ijerph-20-04418-s001.zip › ijerph-2238154-supplementary.pdf]

SUPPLEMENTARY MATERIAL

Strawberry, Blueberry, and Strawberry-Blueberry Blend Beverages Prevent Hepatic Steatosis in Obese Rats by Modulating Key Genes Involved in Lipid Metabolism

Ana María Sotelo-González [1], Rosalía Reynoso-Camacho [1,*], Ana Karina Hernández-Calvillo [2], Ana Paola Castañón-Servín [1], David Gustavo García-Gutiérrez [1], Haiku Daniel de Jesús Gómez-Velázquez [1,3], Miguel Ángel Martínez-Maldonado [4], Ericka Alejandra de los Ríos [5] and Iza Fernanda Pérez-Ramírez [1,*]

1 Chemistry School, Universidad Autónoma de Querétaro, Querétaro 76010, Mexico
2 Unidad Académica Multidisciplinaria Reynosa-Aztlán, Universidad Autónoma de Tamaulipas, Reynosa 88740, Mexico
3 Facultad de Estudios Superiores Cuautilán, Universidad Nacional Autónoma de México, Querétaro 76231, Mexico
4 División de Gastronomía, Instituto Tecnológico Superior de Huichapan, Huichapan 42411, Mexico
5 Institute of Neurobiology, Campus Juriquilla, Universidad Nacional Autónoma de México, Querétaro 76230, Mexico
* Correspondence: rrcamachomx@yahoo.com.mx (R.R.-C.); iza.perez@uaq.mx (I.F.P.-R.)

.

Table S1. Primer sequences, annealing temperature, and NCBI reference sequence.

Transcript	Sequence	Annealing temperature (Tm)	NCBI reference sequence
Fasn	Fw: CCATTTCCATTGCCCTTAGCC<br>Rv: GTAACACATGCTGCTCAAACGA	60 ºC	NM_017332.2
Acaca	Fw: AGGAAGATGGTGTCCGCTCTG<br>Rv: GGGGAGATGTGCTGGGTCAT	56 ºC	NM_022193.2
Acadm	Fw: GCAGAGAAGAAGGGTGATGAA<br>Rv: ACCGCTGACCCATGTTTAG	60 ºC	NM_016986.2
Cpt1	Fw: GATGCTTTGACAGGTGGTTTG<br>Rv: CTTGTGGGCTTAGGGATGTT	65 ºC	XM_039102321.1
Cd36	Fw: CTTGGATGTGGAACCCATAACT<br>Rv: CAGGCCCAGGAGCTTTATTT	60 ºC	XM_039108092.1
Fatp5	Fw: CCATTTCCATTGCCCTTAGCC<br>Rv: AGTCCTGGATGAAATC	60 ºC	XM_008772501.3

Table S2. Urinary polyphenol metabolites and antioxidant capacity of high fat and fructose diet-fed rats supplemented with strawberry, blueberry, and strawberry-blueberry blend beverages.

Tentative identification	Rt (min)	Molecular formula	Expected mass (Da)	Observed mass (Da)	Mass error (ppm)	Adduct	Concentration (µg/mg of creatinine)				
							SD	HFFD	HFFD+SB	HFFD+BB	HFFD+SBB
Benzoic acid metabolites											
Hippuric acid	3.37	$C_9H_9NO_3$	179.0582	178.0504	-3.4285	$[M-H]^-$	0.051 ± 0.007[b†]	0.025 ± 0.004b	0.035 ± 0.006[b†]	0.096 ± 0.012[a†]	0.043 ± 0.010[b†]
Vanillic acid sulfate	3.93	$C_8H_8O_7S$	247.9991	246.9929	4.3453	$[M-H]^-$	ND	ND	0.040 ± 0.004	ND	ND
Hydroxyhippuric acid	4.96	$C_9H_9NO_4$	195.0532	194.0460	0.8356	$[M-H]^-$	ND	ND	0.012 ± 0.002	ND	ND
Cinnamic acid metabolites											
Caffeic acid glucuronide	2.56	$C_{15}H_{16}O_{10}$	356.0743	355.0675	1.3356	$[M-H]^-$	ND	ND	0.002 ± 0.000[a]	0.004 ± 0.001[a]	ND
Ferulic acid sulfate	2.88	$C_{10}H_{10}O_7S$	274.0147	273.0061	-4.8980	$[M-H]^-$	ND	ND	0.194 ± 0.082[a]	0.132 ± 0.020[a]	0.044 ± 0.004[a]
Coumaric acid sulfate	3.12	$C_9H_8O_6S$	244.0042	242.9963	-2.4014	$[M-H]^-$	ND	ND	0.022 ± 0.002	ND	ND
Caffeic acid sulfate	3.80	$C_9H_8O_7S$	259.9991	258.9917	-0.3509	$[M-H]^-$	ND	ND	0.025 ± 0.002[a]	0.005 ± 0.000[a]	ND
Dihydrocoumaric acid	3.81	$C_9H_{10}O_3$	166.0630	165.0555	-1.2603	$[M-H]^-$	ND	ND	0.116 ± 0.009[a]	0.036 ± 0.007[a]	ND
Coumaric acid	4.23	$C_9H_8O_3$	164.0473	163.0397	-2.2924	$[M-H]^-$	0.015 ± 0.001[b†]	0.005 ± 0.001[e]	0.018 ± 0.001[a†]	0.008 ± 0.001[d†]	0.011 ± 0.002[c†]
Dimethylellagic acid	9.35	$C_{16}H_{10}O_8$	330.0376	329.0318	4.5590	$[M-H]^-$	ND	ND	0.011 ± 0.001	ND	ND
Ellagitannin metabolites											
Dimethylurolithin D sulfate	3.53	$C_{15}H_{12}O_9S$	368.0202	367.0136	1.8186	$[M-H]^-$	ND	ND	0.004 ± 0.001	ND	ND
Methylurolithin D glucoronide	3.77	$C_{21}H_{22}O_{11}$	450.1162	449.1098	2.0200	$[M-H]^-$	ND	ND	0.086 ± 0.0006	ND	ND
Urolithin C	4.71	$C_{13}H_8O_5$	244.0372	243.0300	0.3044	$[M-H]^-$	ND	ND	0.196 ± 0.011	ND	ND
Methylurolithin A	5.18	$C_{14}H_{10}O_4$	242.0579	241.0503	-1.3359	$[M-H]^-$	ND	ND	0.026 ± 0.004	ND	ND
Urolithin B glucuronide	6.62	$C_{19}H_{16}O_9$	388.0794	387.0738	4.1636	$[M-H]^-$	ND	ND	2.910 ± 0.171	ND	ND
Urolithin A	9.32	$C_{13}H_8O_4$	228.0423	227.0347	-1.0854	$[M-H]^-$	ND	ND	8.251 ± 0.496[a]	ND	2.326 ± 0.246[b]
Urolithin B	11.20	$C_{13}H_8O_3$	212.0473	211.0404	1.4379	$[M-H]^-$	ND	ND	5.936 ± 0.416[a]	ND	1.816 ± 0.140[a]
Flavanol metabolites											
(Epi)-catechin glucuronide	1.14	$C_{21}H_{22}O_{12}$	466.1111	465.1033	-1.2718	$[M-H]^-$	ND	ND	ND	0.013 ± 0.000[a]	0.010 ± 0.004[a]
Methyl-(epi)-catechin glucuronide	2.94	$C_{22}H_{24}O_{12}$	480.1268	479.1196	0.2521	$[M-H]^-$	0.002 ± 0.000[ab†]	0.002 ± 0.000[b]	0.044 ± 0.007[ab†]	0.055 ± 0.004[a†]	0.051 ± 0.006[a†]
Flavonol metabolites											
Dimethyl quercetin	11.12	$C_{17}H_{14}O_7$	330.0740	329.0681	4.2260	$[M-H]^-$	0.312 ± 0.025[a†]	0.197 ± 0.034[ab]	0.313 ± 0.022[a†]	0.084 ± 0.007[b†]	0.212 ± 0.019[ab]
Flavone metabolites											
Apigenin diglucuronide	2.94	$C_{27}H_{26}O_{17}$	622.1170	621.1094	-0.5710	$[M-H]^-$	0.027 ± 0.004[a†]	0.005 ± 0.002[b]	0.024 ± 0.002[ab†]	0.026 ± 0.002[a†]	0.026 ± 0.002[ab†]
Apigenin glucuronide	4.44	$C_{21}H_{18}O_{11}$	446.0849	445.0757	-4.3859	$[M-H]^-$	7.378 ± 0.570[ab]	6.371 ± 1.234[b]	9.137 ± 0.298[a†]	6.744 ± 1.075[b]	7.766 ± 0.909[ab†]
Gallic acid metabolites											
Methylpyrogallol sulfate	3.77	$C_7H_8O_6S$	220.0042	218.9965	-1.7285	$[M-H]^-$	ND	ND	0.006 ± 0.001	ND	ND
Isoflavone metabolites											
Dihydrodaidzein glucuronide	4.24	$C_{21}H_{20}O_{10}$	432.1056	431.0965	-4.2433	$[M-H]^-$	0.403 ± 0.022[b]	0.379 ± 0.099[b]	0.480 ± 0.020[ab]	0.531 ± 0.090[a†]	0.483 ± 0.031[ab]
Dihydrodaidzein	4.67	$C_{15}H_{12}O_4$	256.0736	255.0664	0.5466	$[M-H]^-$	0.014 ± 0.001[a†]	0.005 ± 0.001[b]	0.013 ± 0.001[a†]	0.011 ± 0.002[ab†]	0.013 ± 0.002[ab†]
Genistein glucuronide	5.20	$C_{21}H_{18}O_{11}$	446.0849	445.0760	-3.7303	$[M-H]^-$	0.631 ± 0.020[a]	0.473 ± 0.093[ab]	0.437 ± 0.015[ab]	0.416 ± 0.054[ab]	0.432 ± 0.020[b]
Glicytein glucuronide	5.52	$C_{22}H_{20}O_{11}$	460.1006	459.0916	-3.7453	$[M-H]^-$	2.374 ± 0.763[ab]	2.553 ± 0.356[b]	2.680 ± 0.278[ab]	3.064 ± 0.374[ab†]	3.169 ± 0.131[a†]
Equol glucuronide	5.69	$C_{21}H_{22}O_9$	418.1264	417.1173	-4.3003	$[M-H]^-$	12.105 ± 0.583[a]	9.953 ± 1.917[a]	10.587 ± 0.691[a]	9.586 ± 1.650[a]	9.520 ± 1.838[a]
Hydroxyequol	8.13	$C_{15}H_{14}O_4$	258.0892	257.0808	-4.3725	$[M-H]^-$	0.018 ± 0.001[a†]	0.011 ± 0.001[b†]	0.015 ± 0.003[ab†]	0.013 ± 0.001[ab†]	0.026 ± 0.040[ab†]
Hydroxyglycitein	8.59	$C_{16}H_{12}O_6$	300.0634	299.0552	-3.0659	$[M-H]^-$	0.042 ± 0.002[a†]	0.032 ± 0.003[b]	0.037 ± 0.002[ab†]	0.038 ± 0.003[ab†]	0.034 ± 0.004[b]
Hydroxygenistein	9.16	$C_{15}H_{10}O_6$	286.0477	285.0396	-2.9990	$[M-H]^-$	0.011 ± 0.001a[†]	0.008 ± 0.001[b]	0.008 ± 0.001[ab]	0.011 ± 0.002[a]	0.011 ± 0.002[ab†]
Equol	11.22	$C_{15}H_{14}O_3$	242.0943	241.0862	-3.3874	$[M-H]^-$	0.051 ± 0.003[a†]	0.032 ± 0.003[b]	0.037 ± 0.002[ab]	0.040 ± 0.005[ab†]	0.037 ± 0.004[b†]
Methylequol	11.42	$C_{16}H_{16}O_4$	272.1049	271.0976	0.1402	$[M-H]^-$	0.004 ± 0.001[a†]	0.003 ± 0.000[b]	0.003 ± 0.000[ab†]	0.004 ± 0.001[ab†]	0.003 ± 0.000[a†]
Hydroxydaidzein	11.62	$C_{15}H_{10}O_5$	270.0528	269.0451	-1.7580	$[M-H]^-$	12.948 ± 1.043[bc]	11.437 ± 2.001[c]	12.847 ± 1.431[bc]	14.747 ± 2.123[b†]	17.477 ± 1.764[a†]
Lignans											
Enterodiol	9.09	$C_{18}H_{22}O_4$	302.1518	301.1458	4.1634	$[M-H]^-$	ND	ND	0.016 ± 0.001[ab]	0.007 ± 0.003[b]	0.027 ± 0.003[a]
Enterolactone sulfate	9.89	$C_{18}H_{18}O_7S$	378.0773	377.0705	1.2812	$[M-H]^-$	0.234 ± 0.006[ab]	0.153 ± 0.013[ab]	0.349 ± 0.018[ab]	0.084 ± 0.011[b]	0.102 ± 0.009[ab]

Enterolactone	11.18	$C_{18}H_{18}O_4$	298.1205	297.1132	-0.1737	$[M-H]^-$	$4.262 \pm 0.193^{ab\dagger}$	2.983 ± 0.300^{b}	3.120 ± 0.246^{b}	$9.023 \pm 0.443^{a\dagger}$	$7.597 \pm 1.055^{a\dagger}$
Other polyphenols											
Catechol sulfate	2.22	$C_6H_6O_5S$	189.9936	188.9858	-2.8666	$[M-H]^-$	ND	ND	0.305 ± 0.024	ND	ND
Catechol	2.38	$C_6H_6O_2$	110.0368	109.0297	2.1231	$[M-H]^-$	ND	ND	0.012 ± 0.001	ND	ND
Phenylacetic acid metabolites											
Hydroxyphenylacetic acid	2.75	$C_8H_8O_3$	152.0473	151.0394	-4.4361	$[M-H]^-$	ND	ND	0.089 ± 0.007^{a}	0.079 ± 0.008^{a}	ND
Methoxyphenylacetic acid	4.55	$C_9H_{10}O_3$	166.0630	165.0555	-1.0746	$[M-H]^-$	0.154 ± 0.010^{ab}	0.136 ± 0.023^{ab}	$0.37 \pm 0.009^{ab\dagger}$	$0.064 \pm 0.007^{a\dagger}$	$0.268 \pm 0.041^{b\dagger}$
Phenylpropanoic acid metabolites											
Dihydroferulic acid sulfate	3.47	$C_{10}H_{12}O_7S$	276.0304	275.0223	-2.7228	$[M-H]^-$	$0.053 \pm 0.005^{a\dagger}$	0.016 ± 0.005^{d}	0.021 ± 0.003^{cd}	$0.026 \pm 0.003^{c\dagger}$	$0.042 \pm 0.005^{b\dagger}$
Phenylpropionic acid	4.39	$C_9H_{10}O_2$	150.0681	149.0607	-0.4333	$[M-H]^-$	ND	ND	0.030 ± 0.003	ND	ND
Dihydroferulic acid	5.00	$C_{10}H_{12}O_4$	196.0736	195.0662	-0.4924	$[M-H]^-$	ND	ND	0.085 ± 0.006	ND	ND
Phenylvaleric acid metabolites											
Dihydroxyphenylvaleric acid	3.93	$C_{11}H_{14}O_4$	210.0892	209.0823	1.9848	$[M-H]^-$	ND	ND	0.045 ± 0.002^{b}	0.133 ± 0.010^{a}	ND
Dihydroxyphenylvaleric acid sulfate	10.98	$C_{11}H_{14}O_7S$	290.0460	289.0388	0.2431	$[M-H]^-$	$0.027 \pm 0.004^{bcd\dagger}$	0.012 ± 0.004^{d}	$0.054 \pm 0.004^{ab\dagger}$	$0.051 \pm 0.005^{bc\dagger}$	$0.022 \pm 0.003^{cd\dagger}$
Valerolactone metabolites											
Dihydroxyphenylvalerolactone sulfate	3.18	$C_{11}H_{12}O_7S$	288.0304	287.0238	2.3654	$[M-H]^-$	ND	ND	0.259 ± 0.018^{a}	0.212 ± 0.029^{ab}	0.121 ± 0.013^{b}
Dihydroxyphenylvalerolactone	3.24	$C_{11}H_{12}O_4$	208.0736	207.0672	4.3184	$[M-H]^-$	ND	ND	0.055 ± 0.005^{ab}	0.142 ± 0.035^{a}	0.047 ± 0.009^{b}
Methoxyhydroxyphenylvalerolactone	11.43	$C_{12}H_{14}O_4$	222.0892	221.0819	0.0663	$[M-H]^-$	1.898 ± 0.108^{a}	1.745 ± 0.140^{a}	$1.652 \pm 0.059^{ab\dagger}$	$1.201 \pm 0.097^{b\dagger}$	$0.676 \pm 0.039^{b\dagger}$
**Urinary antioxidant capacity**											
Folin-Ciocalteu reducing capacity assay (mg GAE/mg creatinine)							1.2 ± 0.4^{b}	0.8 ± 0.4^{b}	0.8 ± 0.3^{b}	2.3 ± 0.7^{a}	0.9 ± 0.5^{b}
$DPPH^+$ radical scavenging assay (mg TE/mg creatinine)							4.8 ± 2.4^{b}	1.8 ± 0.4^{c}	2.3 ± 0.9^{bc}	8.3 ± 2.4^{a}	3.8 ± 1.8^{bc}
$ABTS^-$ radical scavenging assay (mg TE/mg creatinine)							2.8 ± 1.7^{b}	1.7 ± 0.9^{b}	6.1 ± 3.0^{a}	5.7 ± 2.9^{a}	4.0 ± 2.9^{a}

Data are showed as mean ± standard deviation of ten replicates. Different letters indicate significant ($p<0.05$) differences between samples by Tukey's or Friedman's test. †Indicate significant ($p<0.05$) difference as compared to the HFFD group by Dunnet's or Wilcoxon test. SD: standard diet; HFFD: high fat and fructose diet; SB: strawberry beverage; BB: blueberry beverage; SBB: strawberry-blueberry beverage; DPPH: 2,2-diphenyl-1-picrylhydrazyl; ABTS: 2,2'-azino-bis(3-ethylbenzo-thiazoline-6-sulfonic acid); GAE: gallic acid equivalent; TE: trolox equivalent; Trolox: 6-hydroxy-2,5,7,8-tetramethylchroman-2-carboxylic acid.

Table S3. Top 25 loading values of component 1 from the sparse Partial Least Square-Discriminant Analysis of urinary polyphenol metabolites of high fat and fructose diet-fed rats supplemented with strawberry, blueberry, and strawberry-blueberry blend beverages.

**Urinary metabolites**	**Loadings 1**	
	**Component 1**	**Component 2**
Catechol sulfate	0.2710	0
Urolithin B glucuronide	0.2696	0
Urolithin C	0.2694	0
Dihydroferulic acid	0.2689	0
Methylurolithin A	0.2658	0
Methylpyrogallol sulfate	0.2656	0
Hydroxyhippuric acid	0.2656	0
Caffeic acid sulfate	0.2526	0
Methylurolithin D glucuronide	0.2237	0
Vanillic acid sulfate	0.2207	0
Catechol	0.2197	0
Dimethylellagic acid	0.2189	0
Urolithin A	0.2034	0.0769
Dihydrocoumaric acid	0.1923	0
Hydroxydaidzein	0.1794	0
Phenylpropionic acid	0.1640	0
Dimethylurolithin D sulfate	0.1639	0
Coumaric acid sulfate	0.1601	0
Urolithin B	0.1524	0.0654
Enterolactone	0.1137	0.2547
Hydroxyphenylacetic acid	0.0986	0.0184
Dihydroxyphenylvalerolactone sulfate	0.0450	0.2139
Enterolactone sulfate	0.0249	0.0526
Ferulic acid sulfate	0.0087	0.1636
Methoxyphenylacetic acid	0.0041	0

Data was normalized by sum, square root transformed, and auto scaled.

Table S4. Top 25 loading values of component 2 from the sparse Partial Least Square-Discriminant Analysis of urinary polyphenol metabolites of high fat and fructose diet-fed rats supplemented with strawberry, blueberry, and strawberry-blueberry blend beverages.

**Urinary metabolites**	**Loadings 1**	
	**Component 1**	**Component 2**
Methyl-(epi)-catechin glucuronide	0	0.4208
Methoxyhydroxyphenylvalerolactone	0	0.3746
Equol glucuronide	0	0.3099
Dimethyl quercetin	0	0.3034
Dihydroxyphenylvalerolactone	0	0.2936
Hydroxyglycitein	0	0.2594
Enterolactone	0.1137	0.2547
(Epi)-catechin glucuronide	0	0.2362
Enterodiol	0	0.2338
Dihydroxyphenylvalerolactone sulfate	0.0450	0.2139
Apigenin glucuronide	0	0.2123
Ferulic acid sulfate	0.0087	0.1636
Dihydroxyphenylvaleric acid	0	0.1463
Dihydrodaidzein glucuronide	0	0.0938
Urolithin A	0.2034	0.0769
Urolithin B	0.1524	0.0654
Glicytein glucuronide	0	0.0545
Enterolactone sulfate	0.0249	0.0526
Caffeic acid glucuronide	0	0.0470
Dihydroferulic acid sulfate	0	0.0448
Dihydroxyphenylvaleric acid sulfate	0	0.0382
Coumaric acid	0	0.0269
Hydroxyphenylacetic acid	0.0986	0.0184
Equol	0	0.0112
Methylequol	0	0.0097

Data was normalized by sum, square root transformed, and auto scaled.

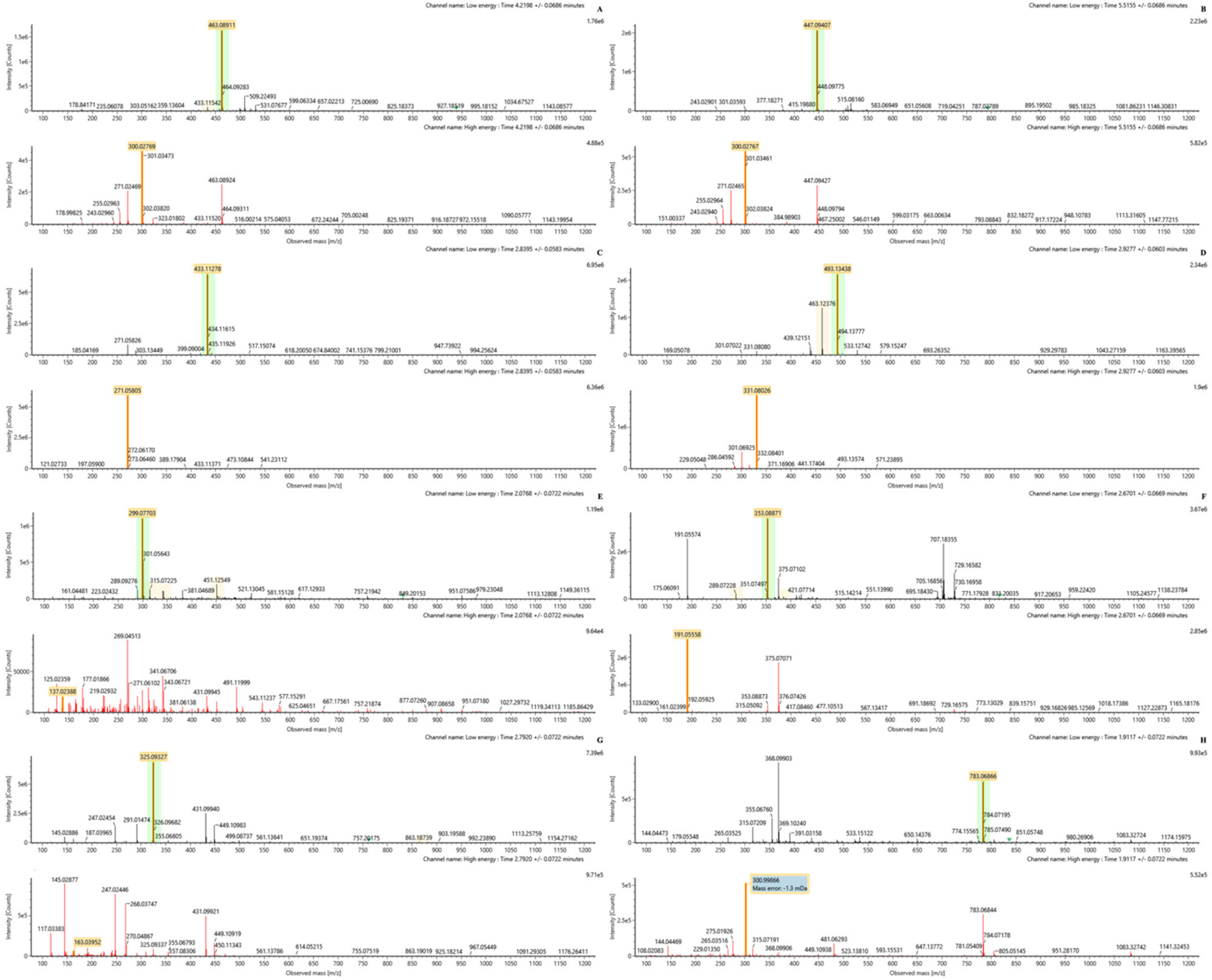

Figure S1. High resolution MS^E spectra at high (superior) and low (inferior) collision energy of the major polyphenols identified in berry fruit beverages: (A) myricetin rhamnoside, (B) quercetin rhamnoside, (C) pelargonidin hexoside, (D) malvidin hexoside, (E) hydroxybenzoic acid hexoside, (F) caffeoylquinic acid isomer II, (G) coumaric acid hexoside, and (H) peduncalagin.
